# Dispersal Limitation Dominates Riverine Fish Communities in the Areas of the Water Diversion Project in the Western Sichuan Plateau, China

**DOI:** 10.3390/ani15050730

**Published:** 2025-03-04

**Authors:** Tao Chang, Zheng Gong, Kunyu Shang, Piao Hu

**Affiliations:** 1 Institute of Hydrobiology, Chinese Academy of Sciences, Wuhan 430072, China; changtao@ihb.ac.cn (T.C.); shangkunyu@ihb.ac.cn (K.S.); hupiao@ihb.ac.cn (P.H.); 2 College of Life Sciences, Zaozhuang University, Zaozhuang 277160, China

**Keywords:** water diversion, fish communities, dispersal limitation, spatial differentiation

## Abstract

The riverine fish species are highly vulnerable and responsive to large-scale water diversion projects. These adverse impacts are more pronounced in the plateau river ecosystems, which may change the environmental conditions of fish habitats and community structure. We investigated the effects of various environmental factors on fish diversity in seven rivers of the Western Sichuan Plateau, which is the planned area of China’s South-to-North Water Diversion Project. Twenty-two fish species, including eight exotic species, were collected during September 2023 (Autumn) and May 2024 (Spring). The fish communities exhibited no significant difference between seasons but had prominent variations among different rivers. The heterogeneity of fish communities was significantly and positively correlated with the geographical distance between the sampling sites (based on a projected coordinate system). Furthermore, the canonical correspondence analysis (CCA) illustrated that altitude contributed more to the distribution of fish species than other physicochemical factors, such as channel width, conductivity, and water temperature. Rivers at low altitudes are likely to be vulnerable to invasion of exotic fish. Our results demonstrated that the dispersal limitation by geographical distance and altitudinal gradient were the primary regulatory factors on the spatial differentiation of fish communities in the rivers of the study area, which reflected a high dependence of fish species on local habitats. As the water diversion project is implemented, more attention is expected to be paid to protecting fish habitats and regime shifts in fish communities. Additionally, the risk assessment of biological invasion under inter-basin water transfers and human activities should be carried out as soon as possible.

## Simple Summary

Human activities can significantly impact the fish diversity in plateau rivers. Understanding the mechanisms underlying community maintenance is crucial for fish conservation. This study investigated the spatiotemporal distribution patterns of fish diversity in seven rivers of the Western Sichuan Plateau, which is the planned area of China’s South-to-North Water Diversion Project. Through a comprehensive analysis of the effects of various environmental factors on fish community variations, we revealed that dispersal limitation served as the primary driver in maintaining fish diversity in plateau rivers. The spatial differentiation of fish communities is predominantly regulated by geographical distance and altitudinal gradient. In particular, low-altitude rivers might be vulnerable to exotic fish invasions. These findings implied that habitat protection should be prioritized in conservation strategies of fish diversity in plateau rivers.

## 1. Introduction

The plateau rivers refer to the rivers that originate from plateaus or mainly flow through plateau regions [1]. They are usually the water sources of many large drainage basins and play a vital ecological role in water resource conservation [2], which supplies more than one-third of the world’s population [3]. Due to the unique environmental conditions, high habitat heterogeneity, and low human activity disturbance, the plateau rivers are refuges for many fish species and the “center of origin” for newly evolved species [4,5]. They are also viewed as the hotspots for global fish diversity research and protection. From the perspective of biological evolution and biogeographical history, the plateau rivers are home to fish groups that can adapt to high altitude, low water temperature, low dissolved oxygen, and torrential flows, such as subfamily Schizothoracinae and genus *Triplophysa* on the Tibetan Plateau [6,7]. However, the harsh environmental conditions also make plateau river ecosystems sensitive and weak in self-regulation ability, resulting in low fish species richness and abundance and unstable fish community structure [8,9,10]. As global climate change and human activities intensify, the ecological environment of plateau rivers is gradually being affected by the construction of hydropower stations, reservoirs, and inter-basin water transfer projects, consequently leading to water volume reduction, water temperature increase, water quality deterioration, sediment accumulation, and habitat destruction [11,12,13], which directly or indirectly alter the assemblies of fish communities and the ecosystem functions of the plateau rivers.

Understanding the driving mechanisms of biodiversity and community structure is central to fish conservation in plateau rivers. Many studies have suggested that environmental filtering and dispersal limitation are the two main mechanisms driving riverine fish community composition [14,15]. The environmental filtering hypothesis establishes that harsh conditions act as filters, allowing the persistence of species necessary to tolerate them [16]. When species have similar ecological preferences, the dispersal limitation acts as the primary driver and geographical distance plays a crucial role in fish spatial distribution [17]. Generally, environmental filtering is more prominent in areas with high environmental variations, such as the floodplain in the middle and lower reaches of the river [18,19,20]. For the fish diversity in plateau rivers, their distribution may vary depending on local habitat and more likely be driven by dispersal limitation [21,22]. Additionally, the effect of environmental filtering also can not be ignored. The ecological environments of plateau rivers can create unique habitats suitable for certain fish species, leading to a more specialized and underdiversified fish community [23]. However, this geographical isolation and environmental stability would be broken by the interbasin water transfers, which could facilitate bidirectional fish movement and further change the fish diversity pattern [24].

Large-scale interbasin water diversion projects have been developed worldwide to alleviate water scarcity problems [25,26]. While supporting regional socioeconomic developments, these projects can also trigger potential ecological impacts on the freshwater ecosystems [27,28]. The South-to-North Water Diversion Project (SNWDP) of China is the world’s most extensive interbasin water transfer program. It has been designed to optimize the allocation schemes of the limited water resources in China and mitigate water scarcity in the arid north and northwest regions. The SNWDP joins the nation’s four major rivers and comprises three transfer lines: the Eastern, Middle, and Western Routes. All these routes draw water from the Yangtze River and deliver it to the Huaihe River, the Yellow River, and the Haihe River, respectively. After the SNWDP implementation, it will form a new water resource network and water supply system for China [29]. Until now, the Eastern and Middle Routes have been completed, but the Western Route is still at the proposal stage because of its complex geological condition and massive water-transfer requirement [30]. The Western Route transfers water from the rivers of the Western Sichuan Plateau, and the areas where the transferred water reaches are typical plateau river ecosystems. It is covered mainly by permafrost and has less resistance to external interference [31,32,33]. Despite harsh climatic conditions, rarity and endemism are very high. According to the list of key protected wild animals from the Sichuan Provincial Department of Ecology and Environment (http://www.scaepp.cn, accessed on 25 September 2024), more than 40 aquatic wild animals are listed as national or provincial protected species in the Western Sichuan Plateau. The protected fish species, such as *Euchiloglanis kishinouyei*, *Gymnodiptychus pachycheilus*, *Schizothorax longibarbus*, and *Schizothorax davidi*, are all recorded in the water-transferred reaches. Because of this fragile ecosystem structure, the Western Route would profoundly impact the aquatic organisms of local plateau rivers.

Most studies have pointed out the eco-environmental change before and after the construction of the Eastern and Middle Routes. For example, the operation of the Middle Route caused water quality deterioration and intensification of the algal bloom. These are mainly attributed to the decreased self-purification capacity of water-transferred reaches due to reduced water flow [34,35]. Guo et al. [36,37] found that the water quality change in the Eastern Route had effects on the fish community assembly. The water clarity (e.g., total suspended solids) and nutrient loading (e.g., phosphate) contributed the most to the variations in fish communities. However, the research referring to Western Route is focused on climate influence [38], water and energy security [39], and flow regime change [40]. Less attention was devoted to aquatic biocenosis, which makes it difficult to predict the potential disturbance impact. Fish diversity is usually regarded as the biological indicator of the river ecosystem health [41] and biological integrity [42] because of its quick response to the environmental changes in river habitats. The Western Route project would alter the river environments and future species distribution patterns. Hence, revealing the interrelation between the fish communities and their habitat physicochemical factors is the key to protecting the structure and function of the plateau river ecosystems. In this study, we investigated the fish diversity in the water-transferred reaches and disentangled the environmental influence on fish communities, aiming to provide evidence for the assessment of the safe operation of SNWDP and the implementation of scientifically based mitigations.

## 2. Materials and Methods

### 2.1. Study Area

The Western Route plans to transfer water from seven rivers, and the water-transferred reaches flow through the Western Sichuan Plateau. They are the Yalong River (YR), Daqu River (DAR), Niqu River (NR), Shuangjiangkou reach of Dadu River (SDR, confluence of two rivers), Duke River (DUR), Make River (MR), and Ake River (AR) [30]. The YR, DAR, and NR belong to the Yalong River basin, and the SDR, DUR, MR, and AR belong to the Dadu River basin. The Yalong River and Dadu River basins are the two primary tributaries of the upper Yangtze River. The average altitude of reaches ranges from 2700 to 4000 m, and the average runoff ranges from 0.7 to 16 billion m^3^ [30]. Besides the Shuangjiangkou Dam in the Dadu River, other reaches would newly construct six dams and draw 4 billion m^3^ of water together according to the project planning, accounting for 32–53% of the runoff of seven rivers [30].

### 2.2. Fish Sampling

**Figure 1:**
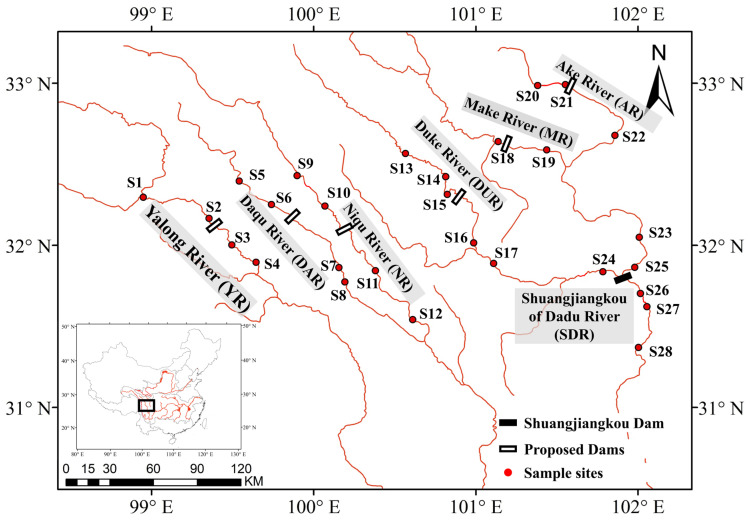
Locations of Shuangjiangkou Dam, proposed dams and fish sampling sites in the seven water-transferred reaches of Western Sichuan Plateau, China. S1: 98°56′57″, 32°17′48″. S2: 99°21′18″, 32°09′58″. S3: 99°29′43″, 32°00′09″. S4: 99°38′41″, 31°53′44″. S5: 99°32′28″, 32°23′45″. S6: 99°44′24″, 32°15′06″. S7: 100°09′21″, 31°51′45″. S8: 100°11′33″, 31°46′26″. S9: 99°53′53″, 32°25′47″. S10: 99°57′49″, 32°21′58″. S11: 100°22′51″, 31°50′36″. S12: 100°36′39″, 31°32′30″. S13: 100°33′58″, 32°33′58″. S14: 100°48′51″, 32°25′25″. S15: 100°49′28″, 32°18′51″. S16: 100°59′12″, 32°00′55″. S17: 101°06′36″, 31°53′19″. S18: 101°08′17″, 32°38′23″. S19: 101°26′13″, 32°35′18″. S20: 101°22′54″, 32°59′07″. S21: 101°33′08″, 32°59′29″. S22: 101°51′28″, 32°40′42″. S23: 102°00′30″, 32°03′00″. S24: 101°47′03″, 31°50′13″. S25: 101°58′48″, 31°51′51″. S26: 102°01′41″, 31°41′58″. S27: 102°03′18″, 31°37′18″. S28: 102°00′13″, 31°22′14″.

Fish species were sampled during September 2023 (Autumn) and May 2024 (Spring). The samples were collected from 2 to 6 sites in each reach (YR = 4, DAR = 4, NR = 4, SDR = 6, DUR = 5, MR = 2, and AR = 3) with a total of 28 sampling sites (Figure 1). The MR had the least sampling sites because of its poor fish diversity under the poaching and hydroelectric exploration, and only two fish species were recorded based on previous research from Wu et al. [43]. To ensure adequate sampling of potentially affected areas, all the sampling sites were deployed upstream and downstream of the planned dams. Nonetheless, our research was focused on the discrepancy of fish communities among the reaches rather than the upstream and downstream. To avoid sampling bias, we captured fish species using the same gillnet set with different mesh sizes at each site (approximately 20 m length, 1.5 m width, 20, 30, and 40 mm mesh sizes). Two fish cages (0.2 × 0.3 × 8 m; mouth opening: 10 × 10 cm; mesh size: 0.1 cm) were used to collect demersal fishes simultaneously. As all studied reaches were shallow (mean water depth < 5 m), the combination of both fishing gears provided sufficient sampling efficiency at different water depths. The nets and cages in each sampling site were set for 18 h (from 4:00 PM to 10:00 AM the next day) to limit the number of fish per net. All specimens were identified to species level, and their body length (standard length in mm) and weight (with a precision of 0.1 g) were measured in situ and released back into the river after measurement.

### 2.3. Physicochemical Factors

During the fish sampling, we synchronously measured physicochemical factors. The water temperature (°C), pH, and conductivity (μS/cm) were measured using a multiparameter water quality analyzer (YSI EXO2). The flow velocity (m/s) was measured using a set of hydrometric propellers. These environmental factors were measured every 100 m with a total of five times at each fish sampling site. The measurement was deployed in a water depth of approximately 1 m and measured 0.5 m below the water surface. The channel width (m) was measured using a diastimeter at the same five sections. The average of the above five environmental measurement data was used for further analysis. The altitude (m) was measured using a portable GPS once at each site because there was no variation in a small spatial scale. All these six factors could be used to characterize the environmental conditions of fish habitats in short-term monitoring.

### 2.4. Data Analysis

The ecological dominance of fish communities was evaluated using the index of relative importance (IRI) [44], which was represented as:

IRI = (N_i_% + W_i_%) × F_i_%

where N_i_ is the individual percentage of certain fish species relative to the total catch, W_i_ is the weight percentage relative to the total catch weight, and F_i_ is the occurrence rate relative to the total sampling sites. The species whose IRI ≥ 1000 are categorized as dominant species, IRI between 100 and 1000 as important species, IRI between 10 and 100 as common species, and IRI values < 10 as rare species.

Seasonal and spatial variation in fish communities in seven water-transferred reaches were determined by Permutational Multivariate Analysis of Variance (PERMANOVA) and Principal Coordinate Analysis (PCoA). The PERMANOVA was used to compare the degree of dissimilarity among groups. The PCoA was conducted to visualize the differences [45,46]. The *p*-values of the aforementioned tests were calculated using 9999 permutations of the procedures.

The geographical distance between the sampling sites was calculated based on the projected coordinate system. The heterogeneity of fish communities between the sampling sites was estimated using the Bray–Curtis dissimilarity index. The geographical distance and community dissimilarity between rivers were represented as the mean values of counterparts for all pairwise sampling sites between rivers. The seasonal differentiation of the Bray–Curtis dissimilarity index was examined by the analysis of variance (ANOVA). The correlation with the significance level between the geographical distance and the community dissimilarity was determined by the Mantel test.

The relationships between fish community structure and physicochemical factors were examined by the canonical correspondence analysis (CCA). The CCA is a constrained principal components analysis of a multiple linear regression-fitted value matrix between the response and explanatory variables matrixes [47]. Before conducting CCA, all the variables were standardized, and the abundance data of each fish species were transformed using the Hellinger transformation to reduce the impact of dominant and rare species on the results. The individual effects with the significance level of each explanatory variable were obtained by hierarchical partitioning (HP) [48]. This statistical approach can solve the multicollinearity among variables, finally obtaining an accurate interpretation for each variable. The contributions of geographical distance and physicochemical factors to the variations in fish communities were determined through variation partitioning analysis (VPA).

All statistical analyses were performed in the R software (Version 3.6.3) using packages “geosphere”, “vegan”, “rdacca.hp”, “picante”, and “ggplot2”.

## 3. Results

### 3.1. Environmental Characteristics

The environmental factors varied among different reaches. The average altitude, channel width, and conductivity of reaches within the Yalong River basin were higher than those in the Dadu River basin. The average water temperature, pH, and flow velocity did not differ significantly between the two basins. Due to increased flooding and air temperature during Autumn, the channel width, water temperature, flow velocity, and conductivity in each reach were slightly higher than in Spring (Table 1).

### 3.2. Species Composition and Dominant Species

**Figure 2:**
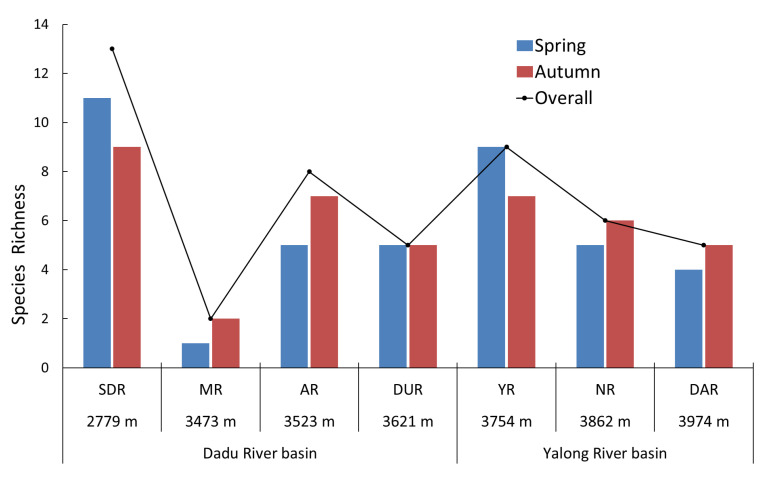
Fish species richness of seven water-transferred reaches in the Western Sichuan Plateau. The black dot line was the overall species richness in each reach. SDR: Shuangjiangkou of Dadu River, MR: Make River, AR: Ake River, DUR: Duke River, YR: Yalong River, NR: Niqu River, DAR: Daqu River.

A total of 22 fish species were collected, with 20 species during the Spring and 19 species during the Autumn, showing no seasonal differencing. The fish species richness of each reach had a considerable variation. The order from high to low was SDR (13 species) > YR (9 species) > AR (8 species) > NR (6 species) > DAR (5 species) > DUR (5 species) > MR (2 species) (Figure 2, Table 2). There were 14 species belonging to endemic species and another 8 species belonging to invasive species. The richness of invasive species was highest in SDR (six species), followed by AR (four species), and was lowest in YR (one species).

The dominant species were *Schizopygopsis chengi* (IRI = 3858) and *Schizopygopsis malacanthus* (IRI = 3025). Additionally, there were 3 important species (100 ≤ IRI < 1000), 10 common species (10 ≤ IRI < 100), and 7 rare species (IRI < 10). No change in dominant species was found between both seasons. The *Schizopygopsis chengi* was dominant in all the reaches within the Dadu River basin (SDR, MR, AR, and DUR), while *Schizopygopsis malacanthus* was dominant in all the reaches within the Yalong River basin (YR, NR, and DAR). The cumulative abundance and biomass of the top five dominant species accounted for 90% and 77% of the total abundance and biomass, respectively (Table 3).

### 3.3. Spatial–Temporal Changes in Fish Communities

**Figure 3:**
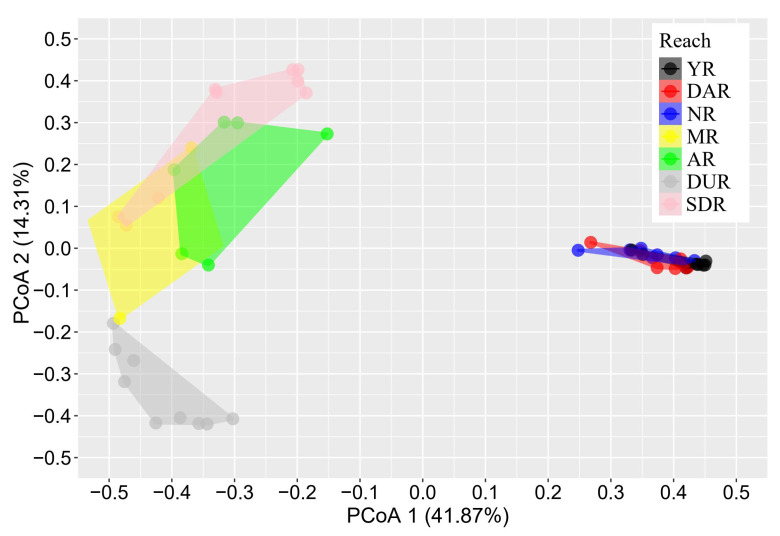
Principal coordinates analysis (PCoA) of fish communities between different water-transferred reaches.

The PERMANOVA and PCoA analyses exhibited that the seasonal change had no significant effect on the fish community structure (*p* = 0.123), but communities among different reaches had profound variation (*p* = 0.001) (Figure 3). The paired test showed that the communities among reaches within the Yalong River basin (YR, NR, and DAR) exhibited no significant difference (*p* > 0.05), while the communities among reaches within the Dadu River basin (SDR, MR, AR, and DUR) had significant differences (*p* < 0.05). The communities between reaches within the Yalong River basin and Dadu River basin also exhibited significant differences (*p* < 0.05) (Table 4).

### 3.4. Influence of Geographical Distance

**Figure 4:**
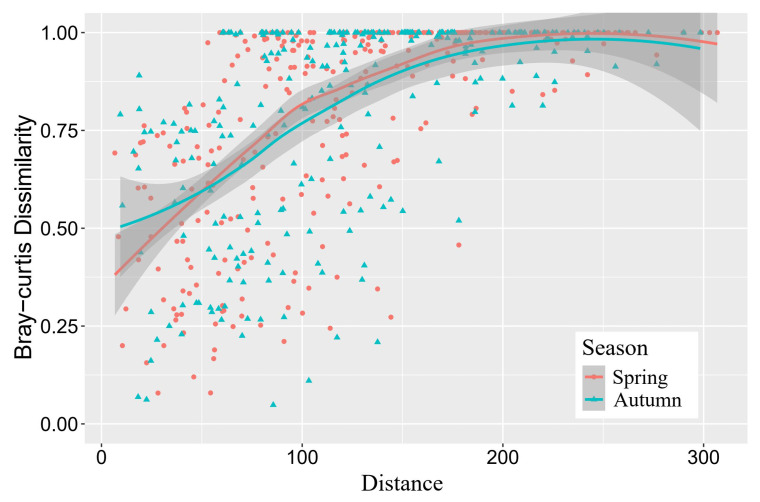
Correlation of geographical distance with the Bray–Curtis dissimilarity index of fish communities between sampling sites in the water-transferred reaches.

The Bray–Curtis dissimilarity index of fish communities among sampling sites had no seasonal variation (ANOVA, *p* > 0.05). In both seasons, the Bray–Curtis dissimilarity index had a significant positive correlation with the distance (Mantel test, *p* < 0.01), indicating that community heterogeneity was increased with the geographical distance (Figure 4). The Bray–Curtis dissimilarity index was the highest between the communities of MR and each reach within the Yalong River basin (YR, NR, and DAR) and lowest among the reaches within the Yalong River basin (YR, NR, and DAR) (Table 5).

### 3.5. Influence of Physicochemical Factors

**Figure 5:**
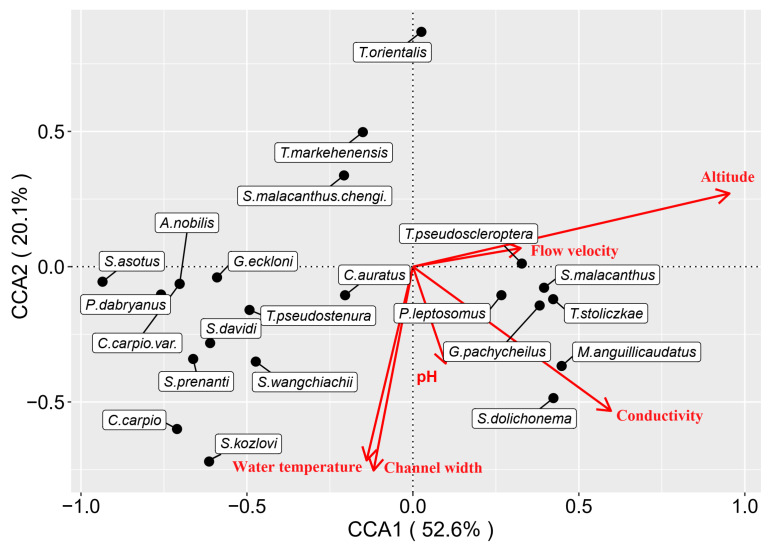
Canonical correspondence analysis (CCA) ordination plots presenting the relationship between fish species and physicochemical factors in the water-transferred reaches.

The CCA ordination plot illustrated the influence of each physicochemical factor on the fish species distribution (Figure 5). It showed that the distribution of *Schizothorax* species (including *S. davidi*, *S. kozlovi*, *S. prenanti*, and *S. wangchiachii*) had a negative relationship to the altitude. *Triplophysa* species (including *T. markehenensis*, *T. pseudoscleroptera*, and *T. stoliczkae*) positively correlated with altitude. Interestingly, almost all the invasive species (including *Aristichthys nobilis*, *Carassius auratus*, *Cyprinus carpio*, *Gymnocypris eckloni*, *Paramisgurnus dabryanus*, and *Silurus asotus*) were captured in low-altitude reaches, such as AR and SDR, inferring their strong invasiveness and habitat preferences to local ecological environments. The HP analysis indicated that altitude, conductivity, channel width, and water temperature significantly impacted the fish distribution (*p* < 0.01). The altitude was the most important environmental variable, which explained 50.1% of the overall variation in fish distribution patterns (Table 6).

### 3.6. Contribution Difference in Different Environmental Factors

**Figure 6:**
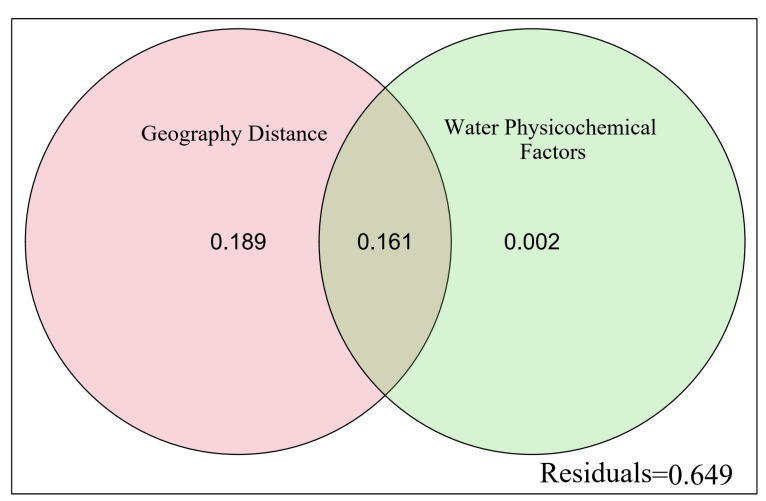
Relative contribution of geographical distance and physicochemical factors on the spatial differentiation of fish communities in the water-transferred reaches.

The VPA showed that the geographical distance and physicochemical factors could explain 35.2% of the total variance in fish communities. The geographical distance contributed 18.9% of the total variance, physicochemical factors contributed 0.2%, and the combined effect was 16.1% (Figure 6). The higher contribution of geographical distance manifested that it was the major driver of fish community variations.

## 4. Discussion

### 4.1. Characteristics of Fish Diversity in Water-Transferred Reaches

The present study is the first full investigation of fish fauna in the areas of Western Route. Only 22 species were collected in the water-transferred reaches, proving a low fish diversity in these plateau rivers. Even so, a high proportion of endemic species (14 species) confirmed that this area was essential for fish diversity conservation. All the endemic species belong to the freshwater fish fauna on the Tibetan plateau [49], and the Schizothoracinae fishes (nine species) were the most abundant. The composition and faunal characteristics of fish diversity were similar to the upper reach of the Yellow River [50] and the Yarlung Zangbo River [51]. Most endemic species only need short-distance migration to breed and feed the periphytic algae and macrozoobenthos [52,53], indicating their admirably well-adaptation and dependence on the plateau river environments. These endemic species usually grow slowly and mature late with low fecundity, making them vulnerable to over-exploitation and sensitive to environmental changes [54,55,56]. If fish stocks are destroyed, recovering over the short-term period would be difficult [57]. Moreover, eight exotic fish were collected in this study, accounting for 36% of the total species. Although their abundance proportion was relatively low (2.4%), measures should be taken quickly to control these invasive species.

The *Hucho bleereri* and *Euchiloglanis davidi* were two national key protected wild fishes recorded in the water-transferred reaches. *Hucho bleereri* was mainly distributed in the Dadu River basin, for which the habitats have suffered a substantial loss in the past 60 years, with a loss rate of 91.4% [58]. *Euchiloglanis davidi* was distributed more widely but has rarely been reported in recent years [59]. Intensive human activities such as hydropower development were deemed to be the main reasons for their habitat loss and population decline. We failed to detect these two species in this survey, demonstrating a further reduction in their population. Our findings revealed that a small number of species accounted for a high proportion of the overall abundance and biomass. This scenario has the potential to exacerbate the precarious status of rare species. Moreover, the dominant species exhibited an increasing trend of concentration, which is detrimental to the stability of the community structure.

### 4.2. Spatio-Temporal Pattern of Fish Communities

No statistical discrepancy was detected in the temporal variation in fish communities. That was because fish species in the water-transferred reaches were sedentary or short-distance migratory species. They did not need large-scale seasonal movement to complete their life history [52,53,59]. We found a significant spatial difference in species richness among the reaches. The SDR, with the lowest average altitude (2779 m), had a higher species richness than other reaches whose average altitude was above 3500 m. Many studies have proven that the altitudinal gradient plays an important role in driving the distribution pattern of biodiversity, which determines many relevant factors, including climate, space, environment, and so on [60]. Jaramillo et al. [61] indicated species richness in streams of the central Andes of Colombia declined rapidly with altitude, and nearly 90% of the species were recorded between 250 and 1250 m. Bhatt et al. [62] uncovered that the richness of fish species in the Himalayan region exhibited a monotonic decline with increasing elevation and reached the peak around mid-elevations (700 to 1500 m). Notably, the fish composition in the SDR was mainly composed of exotic fish (six species) rather than native fish (seven species). It implied that low-altitude reaches are more susceptible to biological invasion, but this potential effect of altitude also could reflect an effect of human population density that tends to be higher at lower elevations.

The distribution discrepancy of native fish suggested their high degree of ecological specialization in local habitats. For example, *Schizopygopsis chengi* was only distributed in the Dadu River basin (SDR, MR, AR, and DUR). *Schizopygopsis malacanthus* was only distributed in the Yalong River basin (YR, NR, and DAR). *Triplophysa markehenensis* was only distributed in the MR. These findings supported the conclusion that the distribution of endemic species represented one of the crucial factors contributing to the spatial differences in plateau riverine fish communities [63,64]. Hence, habitat protection would be paramount in maintaining biological integrity, especially in the plateau rivers.

### 4.3. Dispersal Limitation Determines Spatial Heterogeneity of Fish Communities

The positive correlation between the geographical distance and heterogeneity of fish communities proved that dispersal limitation was the primary driving force in fish community assembly in plateau rivers. The low contribution of physicochemical factors was possibly attributed to the similar river environmental characteristics in the water-transferred reaches, such as high flow velocity, low water temperature, and natural hydrological regime. Li et al. [65] also found that in plateau rivers, spatial heterogeneity of fish communities along elevations is mainly determined by stochastic processes under habitat fragmentation rather than other physicochemical factors. Nonetheless, the altitudinal regulation on species distribution should be valued herein. To be specific, the Schizothoracinae fish can be grouped into three grades, including the primitive grade, specialized grade, and highly specialized grade, which could be distinguished primarily by specialized traits of scales, pharyngeal teeth, and barbels [66]. We found that the primitive grade group (here referring to *Schizothorax*) was concentrated in low-altitude reach (SDR), while the specialized grade group (here referring to *Gymnodiptychus* and *Ptychobarbus*) and highly specialized grade group (here referring to *Schizopygopsis*) were concentrated in high-altitude reaches (DAR and NR). The evolution of Schizothoracinae corresponded to the geological history of the uplift of the Tibetan Plateau. Each genus of Schizothoracinae had its adapted altitude, the geographical altitude of species formation [67,68,69]. Our results emphasized that both horizontal geographical distance and vertical altitude gradient were the determinant factors for fish communities in the study area. That might explain why the combined effect of geographical distance and physicochemical factors was high. Therefore, the spatiotemporal variability and complexity of fish communities in plateau rivers not only relied on river environmental elements but were also affected by the multi-effects of geographical and biological factors such as habitat isolation and species evolutionary history [70,71,72].

The CCA intuitively interpreted the high adaptation of exotic fish in the water-transferred reaches. Most species were eurytopic, omnivorous fish with high tolerance to harsh environmental conditions. They preferred habitats with low altitudes and higher water temperatures (SDR, as mentioned above). These habitats could provide abundant food resources, diversified habitats, and refuges, which is conducive to the survival and competition of exotic fish. A low abundance of exotic fish did not mean a low risk of biological invasion. Liu et al. [73] found that *Pseudorasbora parva* and *Misgurnus anguillicaudatus* had established self-sustaining populations in the middle Yarlung Zangbo River on the Tibetan Plateau. These species had strong invasiveness and negatively affected local fish resources. In addition, the adverse impact of the rising density of the human population in low-altitude areas can not be neglected. The economic and demographic variables reflect the intensity of human activities and integrate the effect of factors that directly determine the outcome of invasion, such as propagule pressure, pathways of introduction, eutrophication, and the intensity of anthropogenic disturbance [74].

## 5. Conclusions

This study revealed a diversified fish species and a high degree of endemism in rivers of the Western Sichuan Plateau. Fish assemblages differed significantly among water-transferred reaches, and the dispersal limitation was regarded as the primary driver shaping the fish communities at the regional scale. Therefore, we highlighted that fish conservation in the plateau rivers should pay more attention to local habitat protection because of the habitat specificity of native species. Additionally, vigilance regarding species endangerment and biological invasion risks will remain indispensable. At present, we have only found exotic fishes in a few reaches. Still, with the implementation of the Western Route, the incessant interference of human activities would heighten the risk of invasion and the dispersal of exotic species. To protect fish diversity in these plateau rivers, it is essential to monitor the dynamics of fish resources, track the invasion pathway of exotic fish, and improve protection measures over time.

## Figures and Tables

**Table 1:** The mean values and seasonal change in physicochemical variables in the seven water-transferred reaches.

Factors	Season	Dadu River Basin				Yalong River Basin		
		SDR	MR	AR	DUR	YR	NR	DAR
Channel width (m)	Spring	98.33	48.44	32.09	49.10	120.79	53.12	50.58
	Autumn	112.54	34.50	43.31	53.84	117.05	56.21	48.48
Water temperature (°C)	Spring	11.08	10.58	8.49	8.04	10.92	10.08	10.03
	Autumn	15.39	10.86	9.49	11.13	11.70	12.17	11.74
pH	Spring	8.22	8.24	7.88	8.22	8.13	8.15	8.17
	Autumn	8.17	5.45	6.78	7.27	8.09	8.55	8.21
Flow velocity (m/s)	Spring	0.53	0.20	0.33	0.47	0.54	0.49	0.39
	Autumn	0.52	0.68	0.52	0.86	0.85	0.75	0.91
Conductivity (μS/cm)	Spring	164.86	173.66	100.49	204.20	230.82	256.20	193.31
	Autumn	223.93	202.90	103.98	223.22	330.95	325.05	214.71
Average altitude (m)		2779	3473	3523	3621	3754	3862	3974

SDR: Shuangjiangkou of Dadu River, MR: Make River, AR: Ake River, DUR: Duke River, YR: Yalong River, NR: Niqu River, DAR: Daqu River.

**Table 2:** List of fish species collected in the seven water-transferred reaches.

Family	Genus	Latin Name	SDR	MR	AR	DUR	YR	NR	DAR
Cyprinidae	*Aristichthys*	*A. nobilis* *	+						
	*Carassius*	*C. auratus* *			+				
	*Cyprinus*	*C. carpio* *	+						
		*C. carpio* var. *	+						
	*Gymnocypris*	*G. eckloni* *	+		+				
	*Gymnodiptychus*	*G. pachycheilus*					+	+	+
	*Ptychobarbus*	*P. leptosomus*	+				+	+	+
	*Schizopygopsis*	*S. chengi*	+	+	+	+			
		*S. malacanthus*					+	+	+
	*Schizothorax*	*S. davidi*	+		+		+		
		*S. dolichonema*					+		
		*S. kozlovi*	+						
		*S. prenanti*	+			+			
		*S. wangchiachii*	+				+		
Nemacheilidae	*Triplophysa*	*T. markehenensis*		+	+				
		*T. orientalis*			+	+			
		*T. pseudoscleroptera*			+	+	+	+	+
		*T. pseudostenura*	+			+		+	
		*T. stoliczkae*					+	+	+
Cobitidae	*Paramisgurnus*	*P. dabryanus* *	+		+				
	*Misgurnus*	*M. anguillicaudatus* *					+		
Siluridae	*Silurus*	*S. asotus* *	+						

SDR: Shuangjiangkou of Dadu River; MR: Make River; AR: Ake River; DUR: Duke River; YR: Yalong River; NR: Niqu River; DAR: Daqu River. Invasive species are indicated with “*”.

**Table 3:** The index of relative importance (IRI) of fish species in the water-transferred reaches, showing the individual percentage (N%), weight percentage (W%) and occurrence rate (F%) of each species.

Species	N%	W%	F%	IRI
*S. chengi*	46.8%	18.2%	59.4%	3858
*S. malacanthus*	30.3%	44.1%	40.6%	3025
*G. pachycheilus*	4.3%	12.6%	40.6%	683
*T. pseudoscleroptera*	5.8%	1.8%	53.1%	404
*T. pseudostenura*	2.9%	0.5%	37.5%	127
*S. prenanti*	2.8%	0.6%	28.1%	95
G. eckloni *	1.6%	1.6%	18.8%	60
*T. stoliczkae*	1.3%	0.3%	34.4%	56
*P. leptosomus*	0.6%	1.6%	21.9%	50
C. carpio *	0.1%	5.7%	6.3%	36
*S. dolichonema*	0.1%	5.0%	6.3%	32
*S. davidi*	0.7%	0.3%	21.9%	22
P. dabryanus *	0.5%	0.4%	15.6%	14
A. nobilis *	0.1%	3.6%	3.1%	11
S. asotus *	0.3%	1.5%	6.3%	11
*T. orientalis*	0.7%	0.2%	9.4%	8
*S. wangchiachii*	0.3%	0.6%	6.3%	5
*C. carpio* var. *	0.0%	1.4%	3.1%	4
*S. kozlovi*	0.2%	0.0%	6.3%	1
C. auratus *	0.2%	0.1%	3.1%	1
M. anguillicaudatus *	0.2%	0.0%	3.1%	1
*T. markehenensis*	0.1%	0.0%	6.3%	1

Invasive species are indicated with “*”.

**Table 4:** The significance testing of spatiotemporal variation in fish communities using permutational multivariate analyses of variance (PERMANOVA) (*: *p* < 0.05).

Region		Sum of Squares	R^2^	F	Pr (>F)
Overall	Season	0.459	0.026	1.509	0.123
	Reaches	10.793	0.621	11.821	0.001 *
	Season and Reaches	0.651	0.037	0.856	0.679
	Residual	5.478	0.315		
	Sum	17.382	1.000		
Reaches	AR vs. DUR	1.499	0.473	11.677	0.002 *
	AR vs. MR	0.121	0.094	0.624	0.590
	AR vs. DAR	2.145	0.527	13.396	0.001 *
	AR vs. NR	2.130	0.495	11.765	0.001 *
	AR vs. YR	2.333	0.589	17.201	0.001 *
	AR vs. SDR	0.983	0.254	4.437	0.001 *
	DUR vs. MR	0.522	0.369	5.258	0.021 *
	DUR vs. DAR	3.300	0.666	29.948	0.001 *
	DUR vs. NR	3.155	0.624	24.852	0.001 *
	DUR vs. YR	3.472	0.719	38.303	0.001 *
	DUR vs. SDR	2.017	0.436	12.355	0.001 *
	MR vs. DAR	1.255	0.523	8.767	0.025 *
	MR vs. NR	1.205	0.463	6.902	0.022 *
	MR vs. YR	1.314	0.607	12.338	0.018 *
	MR vs. SDR	0.566	0.212	2.421	0.055
	DAR vs. NR	0.293	0.120	1.905	0.132
	DAR vs. YR	0.302	0.158	2.619	0.054
	DAR vs. SDR	2.829	0.497	14.814	0.001 *
	NR vs. YR	0.116	0.059	0.874	0.413
	NR vs. SDR	2.609	0.456	12.563	0.001 *
	YR vs. SDR	2.962	0.535	17.281	0.002 *

**Table 5:** Mean geographical distance and Bray–Curtis dissimilarity index of fish communities between different water-transferred reaches.

Reaches	Bray–Curtis Dissimilarity Index		Geographical Distance (Km)	
	Mean	SD	Mean	SD
DAR vs. MR	1.00	0.00	148.11	16.80
NR vs. MR	1.00	0.00	127.96	12.07
YR vs. MR	1.00	0.00	191.93	22.98
SDR vs. DAR	1.00	0.00	198.49	33.39
SDR vs. YR	1.00	0.01	248.52	29.75
YR vs. DUR	0.99	0.02	147.31	26.45
SDR vs. NR	0.99	0.02	169.47	33.68
DAR vs. DUR	0.99	0.03	102.82	23.24
NR vs. DUR	0.99	0.02	83.30	20.75
NR vs. AR	0.96	0.06	168.91	10.39
YR vs. AR	0.96	0.05	231.37	19.95
DAR vs. AR	0.95	0.07	188.89	13.00
SDR vs. DUR	0.84	0.17	117.46	32.31
DUR vs. AR	0.80	0.15	109.00	12.58
SDR vs. AR	0.79	0.16	130.67	28.05
SDR vs. MR	0.79	0.19	115.08	22.16
MR vs. DUR	0.66	0.23	67.98	14.74
NR vs. DAR	0.55	0.20	59.77	33.58
MR vs. AR	0.53	0.24	50.12	9.39
YR vs. DAR	0.49	0.18	65.09	28.98
YR vs. NR	0.48	0.18	93.42	35.20

**Table 6:** Contribution of each physicochemical variable towards the total explained variation in fish-community structure in hierarchical partitioning (HP) analysis. (*: *p* < 0.05).

Variables	Relative Importance (%)	Significance
Altitude	50.1	0.001 *
Conductivity	19.2	0.004 *
Channel width	14.1	0.008 *
Water temperature	9.5	0.025 *
Flow velocity	5.3	0.1249
pH	1.8	0.3506

## Data Availability

The data presented in this study are available on request from the corresponding author.
